# Exploring bacterial and eukaryotic communities in the gut microbiota of urban and rural cats (*Felis catus*) in Colombia

**DOI:** 10.1007/s11259-025-10831-8

**Published:** 2025-09-15

**Authors:** Luisa Páez-Triana, Nicolás Luna, Angie L. Ramirez, Anny Camargo, Ariana Reina, David Cardona, Valeria Velandia, María Fernanda Zúñiga, Luz H. Patiño, Juan David Ramirez, Marina Muñoz

**Affiliations:** 1 Centro de Investigaciones en Microbiología y Biotecnología – UR (CIMBIUR), School of Sciences and Engineering, Universidad del Rosario, Bogotá, Colombia; 2 College of Public Health, University of South Florida, Tampa, FL USA; 3 Departamento de Biología, School of Sciences and Engineering, Universidad del Rosario, Bogotá, Colombia; 4 Instituto de Biotecnología- UN (IBUN), Universidad Nacional de Colombia, Bogotá, Colombia

**Keywords:** Microbiota, Domestic cats, Bacteria communities, Microeukaryote communities, Colombia

## Abstract

Cats are essential companions to humans, offering emotional support while potentially harboring infectious agents. Understanding the gut microbiota of domestic cats (*Felis catus*) is critical for advancing feline health and examining environmental influences on microbial composition. This study utilized 16S and 18S rRNA sequencing to investigate bacterial and eukaryotic communities in the feces of 30 cats from two Colombian regions: urban Bogotá and rural Boyacá. Key bacterial taxa included *Collinsella*, *Bifidobacterium*, and *Alloprevotella* in Bogotá, while *Romboutsia*, *Clostridium* sensu stricto 1, and *Turicibacter* predominated in Boyacá. Similarly, fungal genera such as *Candida* and *Malassezia* were prevalent across all samples, with geographic variations observed for *Blastocystis*, which was more abundant in Bogotá, and *Pseudomonocystis*, which dominated Boyacá. Despite no significant clustering in alpha‑ and beta‑diversity analyses, subtle shifts in the relative abundance of specific taxa were observed and are hypothesized to be influenced by differences in lifestyle and diet, with urban cats tending to remain indoors and consume commercial feeds, while rural cats often scavenge or consume human food. This work also highlights the value of 18S rRNA analysis in characterizing microeukaryotic communities, providing foundational insights into the interactions between feline microbiota and their environments. This study delineates the gut microbiota composition of healthy cats, providing a valuable reference for future feline health research.

**Supplementary Information:**

The online version contains supplementary material available at 10.1007/s11259-025-10831-8.

## Introduction

Companion animals, such as cats (*Felis catus*), have recently played an increasingly important role in human lives. The population of these animals has surged in many cities, making them integral members of households (Bugrov et al. 2022). Their presence has been linked to numerous positive effects, including improved mood, reduced stress, and therapeutic benefits for conditions such as autism and depression (Bugrov et al. 2022; Grajfoner et al. 2021; Taniguchi et al. 2018; Allen 2003). Cats’ companionship has also been shown to enhance the quality of life for the elderly and those suffering from loneliness. Understanding the intestinal microbiota of cats is crucial, as it expands our knowledge of these companion animals. The intestinal microbiota is a complex community of microorganisms, including bacteria, archaea, protozoa, fungi, and viruses (Bugrov et al. 2022). Its role extends beyond digestion, affecting energy homeostasis, metabolism, immune modulation, and developing a resilient epithelium. An imbalance in the microbiota has been associated with diseases in animals and humans, highlighting its importance in health and well-being (Suchodolski 2011; Lyu et al. 2020; Marshall-Jones et al. 2006; Lawley and Walker 2013; Ridlon et al. 2006).

Cats also play a key role in transmitting pathogens to humans, potentially serving as reservoirs for zoonoses (Goldstein and Abrahamian 2015). Transmission routes include inhalation, bites, scratches, and fecal–oral contact. Pathogens like *Campylobacter jejuni*, *Cryptosporidium spp.*, and *Toxoplasma gondii* are significant concerns (Goldstein and Abrahamian 2015). The proximity of cats to humans increases the risk of microbial exchange (Song et al. 2013). Studying the fecal microbiota of cats is, therefore, critical for both feline and human health, especially in children and immunocompromised individuals who are at higher risk of infections.

Research on microbiota in domestic animals has primarily focused on bacterial communities, often overlooking protozoan and fungal communities (Deng and Swanson 2015). However, these microorganisms are integral to the microbiota and serve as pathogens for both cats and humans. Diet, age, environment, and human interaction significantly affect microbial composition (Deng and Swanson 2015). Studies have shown changes in microbiota diversity and abundance related to these factors, particularly in bacterial families such as Bacteroidaceae and Fusobacteriaceae. Additionally, research in mouse models has revealed interactions between intestinal bacteria and *T. gondii*, influencing the immune system.

Given the close relationship between humans and cats, understanding the feline intestinal microbiota is critical for ensuring animal well-being and evaluating its broader ecological and public health implications. This study aims to analyze the bacterial and microeukaryotic gut communities of domestic cats from Bogotá (urban) and Boyacá (rural) in Colombia, employing 16S-rRNA and 18S-rRNA amplicon sequencing. By exploring these microbial communities, we seek to assess how environmental, dietary, and lifestyle factors influence gut microbiota composition and diversity. The findings will provide a foundation for advancing knowledge about the gut microbiome’s role in feline health while emphasizing the significance of microeukaryotic analysis, an often-overlooked aspect of microbiota research. This research also aims to contribute to a better understanding of how geographical and environmental differences shape gut microbiota, fostering insights into the complex dynamics between animal ecosystems and public health.

## Methods

### Sample collection and DNA isolation

**Fig. 1:**
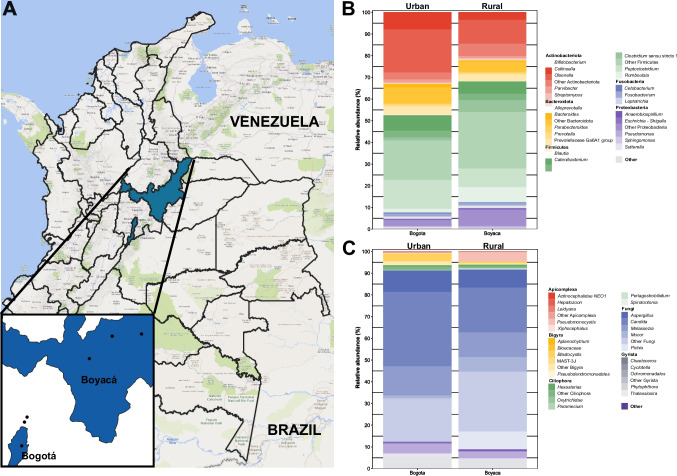
Location and Relative Abundance. **A**. Map of Colombia highlighting Bogotá and Boyacá. Each point represents a geographic location of recollection: Bogotá, Chia, and Cajica, and Tunja, Paipa, and Gameza. **B**. Bacterial communities based on collection department. **C**. Microeukaryotic communities based on collection department

The study included fecal samples from 30 domestic cats in two distinct geographical areas (Fig. 1A). The first area was Bogotá, the capital of Colombia (coordinates 4°36′35″N 74°04′54″W), including the nearby municipalities of Chía and Cajicá, characterized by advanced urban development. Sixteen fecal samples were collected from this urban region. The second area encompassed rural communities in Boyacá Department (coordinates 5°45′N 73°06′W), specifically from the municipalities of Tunja, Gameza, and Paipa, with farming communities and cattle breeders, where 14 fecal samples were collected. In Bogotá, cats were typically indoor pets fed commercial diets, while in Boyacá, cats had more outdoor access and were commonly fed raw or cooked human food or scavenged.

All cats were privately owned and appeared clinically healthy, displaying no signs of disease or recent illness at the time of sampling. As these animals were not presented for clinical care, detailed medical histories and records were not consistently available. Fecal samples were collected with the informed consent of each owner. To ensure sample integrity, fresh feces were collected directly from litter boxes using sterile receptacles, and only the uncontaminated inner portions were used, ensuring each the sample originated from the target individual.

Genetic material was extracted from each fecal sample using the Stool DNA Isolation kit from Norgen (Biotek Corp., Canada). DNA concentration was measured using a Nanodrop spectrophotometer, and integrity was verified via electrophoresis on a 1.5% agarose gel.

### 16S-rRNA and 18S-rRNA amplicon-based sequencing

The DNA samples were sent to an independent entity, Novogene Bioinformatics Technology Co., Ltd, in Beijing, China, for amplicon-based sequencing. This sequencing method focused on the 16S-rRNA and 18S-rRNA V4 hypervariable region, using specific primers (515F and 806R) (Caporaso et al. 2011) and (528F and 706R) (Cheung et al. 2010) to enable the identification of bacteria/archaea and eucaryotic microorganisms, respectively. Library sequencing by end pairing and index adapter ligation were built for each marker and then sequenced on an Illumina NovaSeq 6000 PE250. A minimum expected depth of 100 thousand raw reads was generated for each sample.

We evaluated the quality of the sequencing data by analyzing the raw paired-end demultiplexed sequences with FastQC version 0.11.7 (Leggett et al. 2010) and MultiQC version 1.6 (Ewels et al. 2016). Next, we employed the DADA2 package (Callahan et al. 2016) within R software version 4.0.2 (R Core Team 2021) to filter reads with a Phred score of 30 or higher, infer the amplicon sequence variants (ASVs) using the central sample inference algorithm, merge forward and reverse sequences, and eliminate chimeric structures (Callahan et al. 2016) from the high-throughput sequencing data. In this analysis, we adhered to the recommended parameters for microbiome analysis, as outlined in the DADA2 tutorial (https://benjjneb.github.io/dada2/tutorial.html). Finally, each ASV was taxonomically assigned by comparing it to the SILVA database version 138.1 (Quast et al. 2013) and PR2 database version 5.0.1 (Guillou et al. 2013).

### Prokaryotic and microeukaryotic communities’ analyses

We filtered out amplicon sequence variants (ASVs) associated with mitochondria, chloroplasts, algae, and macroeukaryotic (plants and metazoa) organisms from the abundance and taxonomic assignment tables. This curation process was executed using the R phyloseq package version 1.40.0 (McMurdie et al. 2013). To comprehensively understand the microbial diversity within our samples, we generated rarefaction curves employing the phyloseq (McMurdie et al. 2013) and ampvis2 package version 2.7.31 (Andersen et al. 2018). Furthermore, we standardized our data by rarifying it to ensure consistent sampling depths, thus mitigating the potential impact of variations in sampling depths on dissimilarity metrics (Weiss et al. 2017). This standardization procedure was carried out through the "rarefy_even_depth" function within phyloseq, utilizing a specific seed for the random number generator (rngseed) set to 1 before proceeding with subsequent analyses.

In the domain of microbial community characteristics, we identified the ten most relatively abundant phyla and genera within the cat microbiota. To assess the impact of parameters such as geographical location and toxoplasma detection on the feline microbiota, we conducted non-parametric U Mann–Whitney tests using the stats package version 4.2.0 (R Core Team 2021). For the evaluation of alpha (α) diversity, we quantified the diversity of ASVs using the Shannon–Wiener index (a measure of species diversity) and the Simpson index (a measure of species dominance) from the microbiome package in R version 1.18.0 (L and S 2017). The same non-parametric tests mentioned earlier were employed to analyze the differences observed in the alpha diversity indices. In the context of beta (β) diversity analysis, we assessed and visually represented dissimilarities through principal coordinate analysis (PCoA) using the phyloseq package (McMurdie et al. 2013). This analysis was based on Bray–Curtis distances calculated from the relative abundances of each ASV. Additionally, we applied a permutational multivariate analysis of variance test (PERMANOVA) from the vegan package version 2.6–2 (Oksanen et al. 2013), involving 9,999 permutations, to identify variations in microbiota communities associated with the geographical location and toxoplasma detection.

## Results

Regarding microbial community descriptions, next-generation sequencing allowed us to assign 14,952 ASVs for 16S-rRNA and 3,682 for 18S-rRNA. Among these ASVs, we excluded 142 corresponding to chloroplasts, mitochondria, and eukaryotes and 1,258 corresponding to Metazoa, algae, and dinoflagellates. Finally, 1,955 and 546 ASVs for each respective marker were removed during normalization. The remaining ASVs belong to 58 phyla and 1,043 genera of prokaryotes, 25 subdivisions, and 506 genera of microeukaryotes. Furthermore, rarefaction curves post-normalization (Fig. S1) indicated that the sequencing depth utilized was sufficient for analyzing the diversity and composition of ASVs in each sample. In the context of an amplicon-based sequencing approach targeting the 16S-rRNA region, Firmicutes, Actinobacteriota, Bacteroidota, Proteobacteria, and Fusobacteriota are the five most abundant phyla across all samples (Fig. 1B). Moving to the 18S-rRNA approximation (Fig. 1C), Fungi is the most frequent subdivision across all samples, with genera such as *Candida, Malassezia*, and *Aspergillus* being particularly abundant (~ 80% of relative abundance). Despite their lower abundances (~ 15% relative abundance), we found Apicomplexa, Bigyra, Ciliophora, and Gyrista as part of the most abundant subdivisions.

**Fig. 2:**
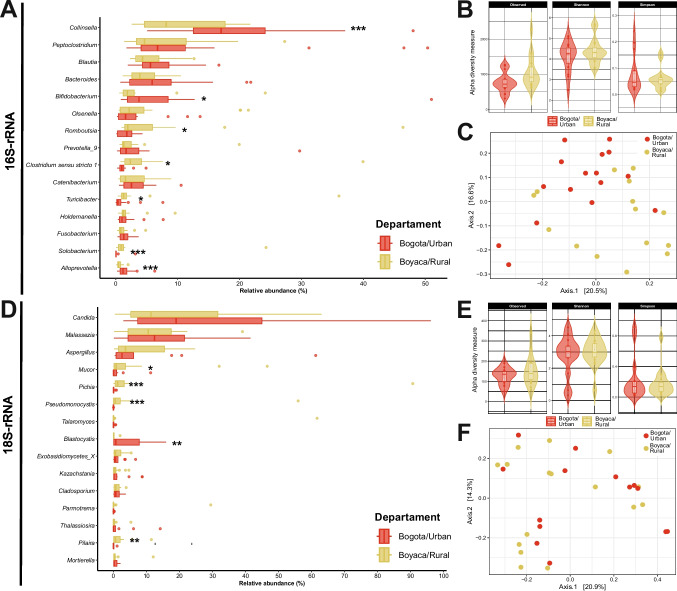
Variation in top 15 genera and Diversity measurements based on departments **A**. Differential abundance of top genera, **B**. Alpha diversity, and **C**. Beta diversity for bacterial communities. **D**. Differential abundance of top genera, **E**. Alpha diversity, and **F**. Beta diversity for bacterial communities * *p* < 0.05, ** *p* < 0.01, *** *p* < 0.001

To assess these potential differences in relative abundance, statistical analyses were conducted on the 15 most abundant genera for both 16S- and 18S-rRNA (Fig. 2). For 16S-rRNA (Fig. 2A), we found variations (*p* < 0.05) concerning the department of origin. Specifically, the genera *Collinsella*, *Bifidobacterium*, and *Alloprevotella* exhibited greater abundance in Bogotá, while *Romboutsia*, *Clostridium* sensu stricto 1, *Turicibacter*, and *Solobacterium* showed higher abundance in Boyacá. Regarding diversity metrics in the samples, specifically the Shannon–Wiener and Simpson indices, they revealed an absence of dominance by any taxonomic group in cat feces. Instead, the diversity was relatively distributed across different taxa. No significant variations were observed between diversities based on department for either the 16S-rRNA (Fig. 2B). In terms of beta diversity analyses using PCoA, a slight separation was noted in the analysis of the 16S-rRNA gene between departments (Fig. 2C).

Concerning 18S-rRNA (Fig. 2D), a parallel pattern emerged, where pronounced changes (*p* < 0.05) were evident based on geographical location (Fig. 2D). Specifically, *Blastocystis* were found in greater abundance in Bogotá. At the same time, *Pseudomonocystis, Mucor, Pichia,* and *Pilaira* exhibited higher abundance in the department of Boyacá. Regarding diversity metrics in the samples, 18S-rRNA (Fig. 2E) didn't show significant variations by the department in alfa diversity and beta diversity (Fig. 2F), where no specific groupings were observed. Statistical examination revealed that neither gene exhibited significant clusters by departments, indicating a random distribution of samples.

## Discussion

Cats, as companion animals, contribute significantly to the well-being of their human owners, not only as beloved pets but also by positively impacting mental and physical health (Bugrov et al. 2022; Grajfoner et al. 2021; Taniguchi et al. 2018; Allen 2003). However, they also play a crucial role in transmitting pathogens like *T. gondii*, a parasite causing severe illness worldwide (Tenter et al. 2000; Furtado et al. 2011). Given their multifaceted significance, research has been initiated on the intestinal microbiota of these pets, aiming to identify and comprehend multiple factors that influence it (Deng and Swanson 2015). This exploration not only seeks to enhance the quality of life for these feline companions but also to unravel potential influences on human health. Utilizing next-generation sequencing techniques, specifically the characterization of bacteria through the 16S-rRNA gene, researchers have described the primary communities under different diets, ages, or environmental factors.

Conversely, the use of 18S-rRNA for deciphering microeukaryotic communities has not been as extensively employed. In this study, we describe bacterial and microeukaryotic communities in the feces of cats from two regions in Colombia. This work aimed to evaluate and formulate hypotheses regarding factors that could potentially determine changes in composition and diversity, such as geographical location.

The analysis revealed the most abundant phyla in the feline microbiota as Actinobacteriota, Bacteroidota, Firmicutes, Fusobacteria, and Proteobacteria (Fig. 1B). These findings align with previous reports indicating the abundance of these phyla in the microbiota of cats, irrespective of their health status, diet, or age (Ritchie et al. 2010; Bermingham et al. 2018; Suchodolski et al. 2015). Particularly noteworthy was the high relative abundance of Firmicutes, consistent with global observations on cat microbiota (Suchodolski 2011; Suchodolski et al. 2015; Tal et al. 2020; Nealon et al. 2023; Tun et al. 2012). Despite identifying bacterial orders consistent with prior studies, their relative abundance varied in this investigation. Conversely, in the realm of eukaryotes, a high abundance of fungi, followed by other parasites like Apicomplexa, was identified (Fig. 1C). The exploration of eukaryotes in the feline intestinal microbiota remains an understudied area. Previous research, primarily focused on fungi, has identified genera such as *Saccharomyces*, *Aspergillus*, and *Penicillium* through the amplification of ITS genes (Handl et al. 2011). While these findings align with the observed dominance of fungi in the cat intestinal microbiota in this study, it is essential to note that using the ITS gene may overlook protozoan communities since this marker is limited to fungi identification. Protozoans, known to interact with the gut microbiota and the immune system, are potentially neglected in these analyses (Denkers 2009; Ulusan Bagci and Caner 2022). The intricate relationship between an unbalanced microbiota affecting protozoan growth or death, and vice versa, highlights the need for a more comprehensive exploration of microeukaryotic communities (Denkers 2009; Ulusan Bagci and Caner 2022).

Furthermore, the presence of organisms such as *Blastocystis* (Fig. 2D), despite not knowing whether they are pathogenic or not, it has been reported to be associated with changes in gene abundance in the human intestinal microbiota (Vega et al. 2021), underscores the importance of delving deeper into microeukaryotic communities in future studies. Also, there are well-established correlations between certain parasites and host intestinal microbiota (Denkers 2009; Ulusan Bagci and Caner 2022). This kind of research can unravel the roles and interactions of fungi, considering the difference in abundance found in previous studies in other companion animals such as dogs (Suchodolski 2011; Suchodolski et al. 2008). But also identify medically significant parasites and elucidate their potential impact on the feline intestinal microbiota.

Visual differences were noted in geographic locations (Fig. 1A and B), and significant variations in relative abundance were observed based on the collection department (Fig. 2), dictated by the distinct environmental settings where the samples were acquired—one being an urban area and its vicinity (Bogotá), and the other comprising municipalities within a department (Boyacá). Our hypothesis based on the limited available data posits that these location-specific changes primarily stem from potential differences in diet and lifestyle, factors influenced by the inhabitants of each region. Cats in urban areas are likelier to remain indoors, refrain from hunting or consuming unfamiliar foods, and predominantly subsist on kibble. Conversely, cats in municipalities tend to have more freedom being less supervised and often have access to consume human food, either raw or cooked. This hypothesis gains support from observed alterations in previous studies. For instance, an increase in the abundance of *Collinsella* and *Bifidobacterium* has been noted in cats fed kibble (Young et al. 2016; Kerr et al. 2014), mirroring the greater abundance observed in the city in this study. Other reports indicate that *Clostridium* can proliferate in diets high in protein and low in carbohydrates in dry food or with raw food, aligning with the increased abundance found in Boyacá.

Furthermore, the genus *Turicibacter* has been linked to diets rich in mixed insoluble fibers and gastrointestinal diseases in cats, including inflammatory bowel diseases—consistent with the lifestyle of Boyacá cats, which exhibited a greater abundance of this genus. The findings presented here, including changes in abundance, limited grouping by beta diversity, and the absence of alterations in alpha diversity, resonate with a previous study involving similar cats, comparing stray cats with owned cats (Ahmad et al. 2021). However, to validate this theory, future studies must effectively control for other variables or collect relevant information from cat owners. Additionally, changes in abundance reported here for genera like *Romboutsia*, *Solobacterium*, and *Alloprevotella* have not been replicated in prior research related to age or diet. Thus, further investigation is imperative to unravel the functionalities of these organisms within the microbiota and comprehend the underlying reasons for changes in abundance.

Nevertheless, multiple genera known to be influenced by the diet of cats (Pilla and Suchodolski 2021) remained unaltered. For instance, *Blautia*, which typically increases in response to mixed insoluble fibers, high-protein, and canned diets, maintained its abundance (Hooda et al. 2013). Similarly, *Bacteroides*, known to decrease in response to canned food or mixed dissoluble fibers (Bermingham et al. 2013; Wernimont et al. 2019), and *Prevotella*, which increases in raw food and decreases in Kibble (Butowski et al. 2019), exhibited no significant changes. Building on these findings, the modest microbiota differences observed in this study appear to fall within the expected range for healthy cats. This aligns with Sung et al.’s work, which demonstrated that healthy felines exhibit relatively invariant gut microbiota over time, in stark contrast to the pronounced disruptions seen in subjects with chronic enteropathy or those undergoing antibiotic treatment (Sung et al. 2024). This comparison strengthens the clinical validity of our results, indicating that the variations we detected likely represent normal temporal fluctuations rather than disease-associated dysbiosis. It underscores the importance of distinguishing between baseline microbiome variability in healthy individuals and changes signaling underlying pathology.

This study provides an initial characterization of the bacterial and microeukaryotic communities in the gut microbiota of urban and rural cats in Colombia. While the absence of complete metadata limited our ability to link observed microbial shifts to specific factors, our findings highlight potential influences of geographic and lifestyle differences on microbial composition. However, our study faced the following limitations: i) Incomplete metadata, such as diet, medication usage, age, breed, lifestyle and other factors, which limited the ability to assess their influence on feline gut microbiota composition. ii) Low detection of protozoan reads, organisms crucial to both feline and human health, which may stem from using broad-target 18S‑rRNA primers for microeukaryotes. iii) A modest sample size per region, which may have hindered the detection of significant variations in microbial diversity and reduced statistical power in beta-diversity analyses. We recommend that future investigations include larger cohorts and detailed metadata collection (diet, medications, age, breed,) to improve interpretation and reproducibility. Despite these limitations, subtle changes in the relative abundance of specific genera, such as the increased presence of *Collinsella* and *Blastocystis* in urban cats and *Romboutsia* and *Pseudomonocystis* in rural ones, provide a foundation for exploring the interplay between diet, environment, and microbiota. These findings underscore the importance of further investigations with larger sample sizes and comprehensive metadata to deepen our understanding of feline microbiota and its implications for health and zoonotic risks. By integrating bacterial and microeukaryotic data, this work highlights the value of combining 16S- and 18S-rRNA analyses for a holistic view of gut ecosystems.

## Supplementary Information

Below is the link to the electronic supplementary material.

Supplementary file1 (PDF 124 KB) Rarefaction curves of 16S and 18S rRNA
