# Supplementary figures and images for: Exploring bacterial and eukaryotic communities in the gut microbiota of urban and rural cats (Felis catus) in Colombia
Source: Vet Res Commun. 2025 Sep 15;49(6):312. doi: 10.1007/s11259-025-10831-8 (PMC12436569; doi:10.1007/s11259-025-10831-8)

**A**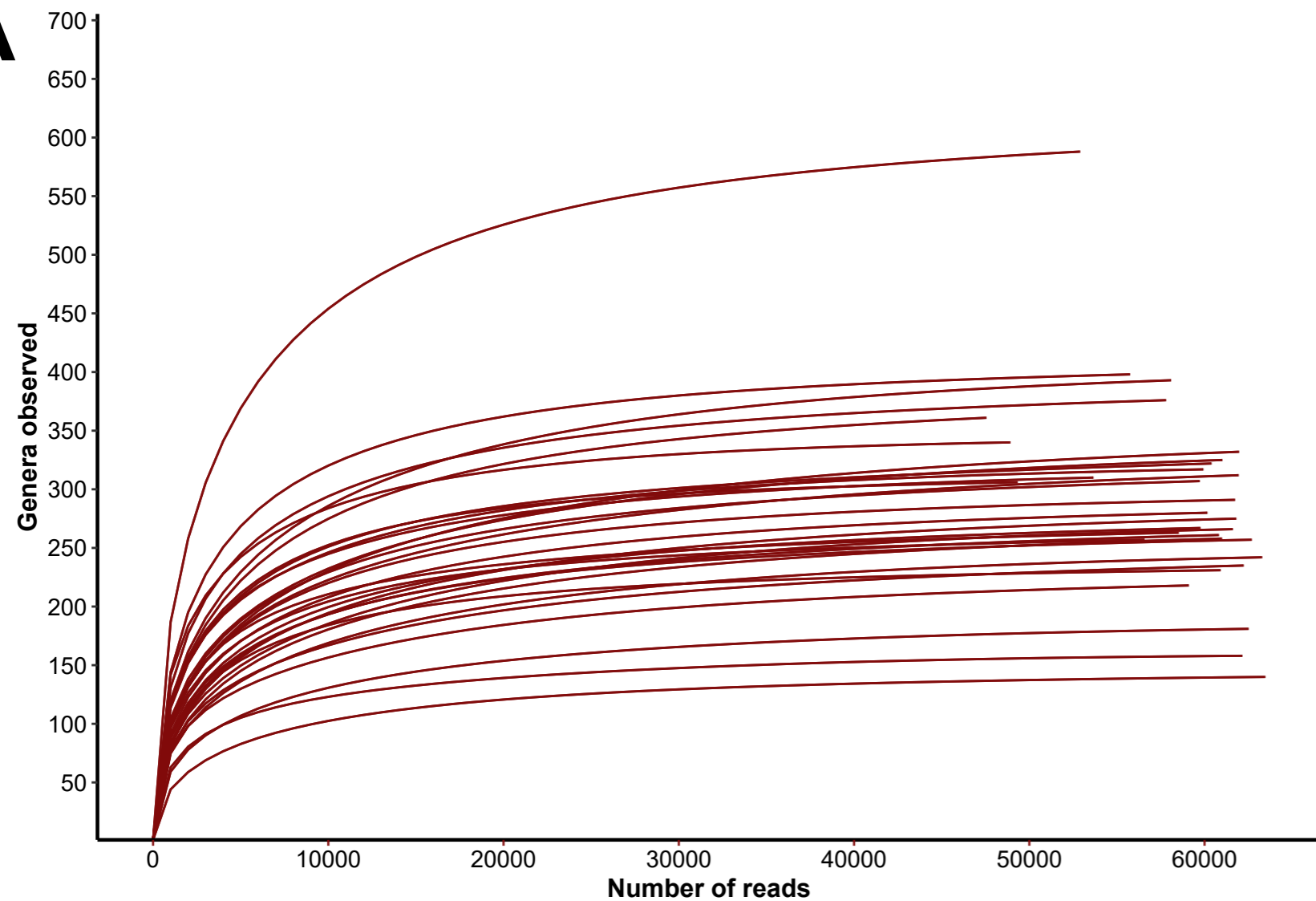**B**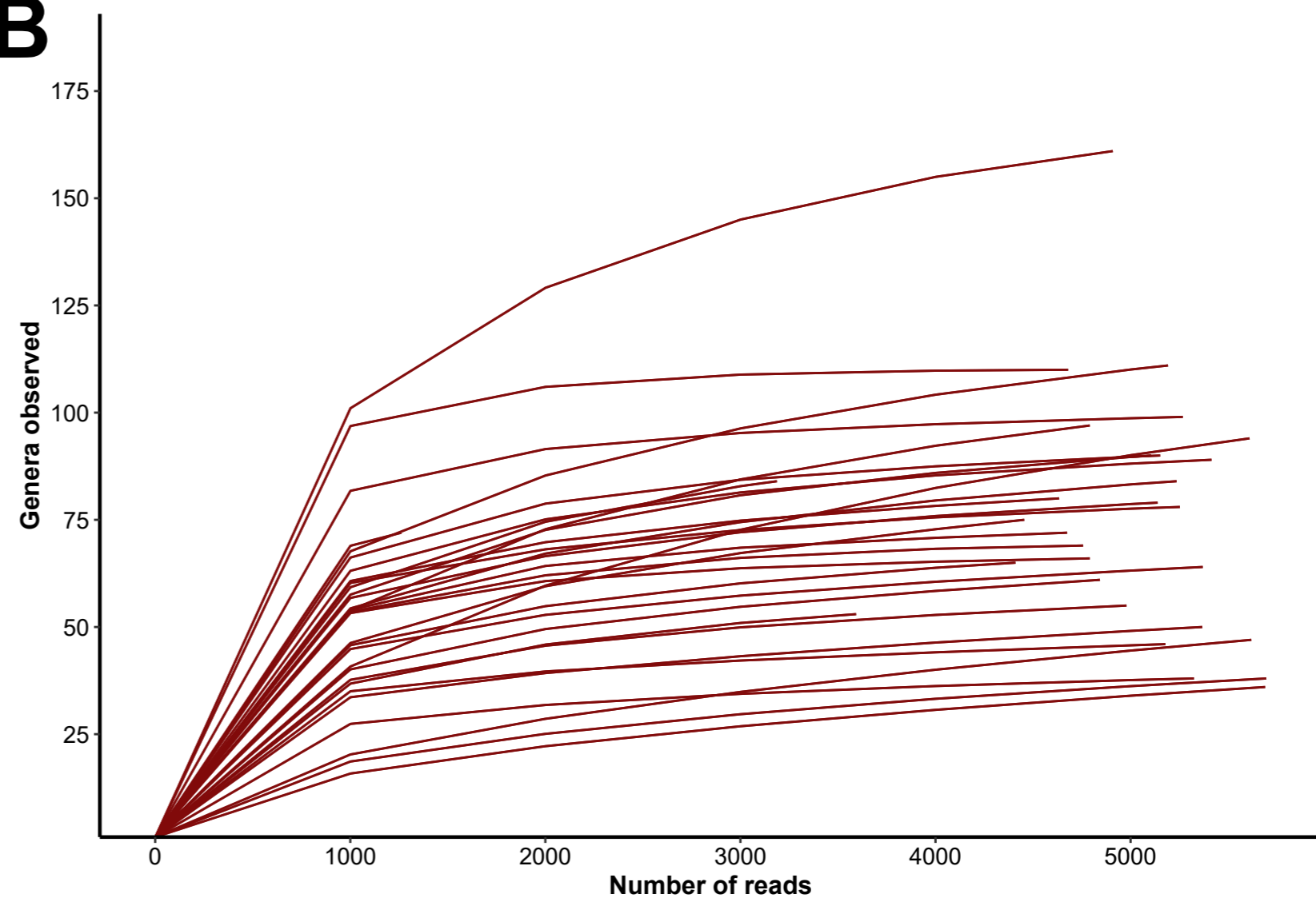

Supplement: Supplementary file 1 — Supplementary file1 (PDF 124 KB) Rarefaction curves of 16S and 18S rRNA [file 11259_2025_10831_MOESM1_ESM.pdf]
